# Chinese anticipate their country’s future to be as bright as their personal future

**DOI:** 10.3389/fpsyg.2026.1704285

**Published:** 2026-02-06

**Authors:** Qi Wang, Nazike Mert, Yi Cao, Yubo Hou

**Affiliations:** 1 Cornell University, Ithaca, NY, United States; 2 Stetson University, DeLand, FL, United States; 3 Peking University, Beijing, China

**Keywords:** Chinese participants, collective future thinking, optimism, perceived control, personal future thinking, valence, wellbeing

## Abstract

Studies on future thinking in primarily Western populations have generally revealed a positivity bias toward the personal future and a negativity bias toward the nation’s future. The present research examined future thinking among Chinese, where college students (Study 1) and community adults (Study 2) were randomly assigned to a personal or a national future condition, where they imagined specific future events that might happen to them or their country in 1 week, 1 year, and 10–15 years. Participants rated the emotional valence and perceived control of each event and completed a wellbeing measure. As expected, Chinese participants imagined personal and national events similarly positive across all time points and perceived similar levels of control for the events. The positivity of personal future events and distant national future events was associated with psychological wellbeing. These findings shed new light on the anticipatory processes in personal and collective future thinking.

## Introduction

A bright future gives us hope and motivates us to work towards our goals. Unsurprisingly, individuals, within the normal population, generally exhibit a positivity bias towards the personal future, expecting more pleasant than unpleasant events to happen in their future lives (Jeon et al., 2020; Newby-Clark and Ross, 2003; Stephan et al., 2015). This positivity bias in personal future thinking is further associated with enhanced psychological wellbeing (Hallford and D’Argembeau, 2022; Hazan et al., 2024; MacLeod, 2025; MacLeod and Conway, 2007; Wang and Koh, 2015). However, recent research has shown that the optimism dissipates when individuals think about the nation’s future: Not only do they expect more unpleasant than pleasant events to happen in the future of their country, but they also imagine their country’s future to be more negative than their personal future (Shrikanth et al., 2018; Shrikanth and Szpunar, 2021). This negativity bias in collective future thinking—a disassociation from the positivity bias in personal future thinking—has been observed in people from many Western societies such as the US, Canada, France, and Britian as well as some non-Western societies like Turkey (Ionescu et al., 2023; Mert and Wang, 2024; Shrikanth and Szpunar, 2021; Yamashiro et al., 2023), raising concerns for its implications for critical issues ranging from mental health to civic engagement to national and international policies (Liu and Szpunar, 2023; Wang and Mert, 2025).

## The unique pattern of collective future thinking in Chinese

The negativity bias in collective future thinking appears to be non-universal, however. Extant research has shown that Chinese participants anticipated predominantly positive events to happen in the nation’s future (Mert et al., 2023; Yao et al., 2025). They also think positively about things to happen in their country’s future across most of the timeliest national and global issues, such as economy, health care, education, and climate change (Mert and Wang, 2025). In the only study to date in which Chinese participants were asked to imagine both personal and national events in the future (Deng et al., 2023), they expected their country’s future to comprise similarly pleasant and unpleasant events as their personal future, thus showing an absence of the disassociation between personal and collective future thinking observed among Western participants (Deng et al., 2023; Mert and Wang, 2024; Shrikanth et al., 2018; Shrikanth and Szpunar, 2021). This unique pattern of findings pertaining to collective future thinking in Chinese participants has been attributed to a strong national identity and a general satisfaction with the overall condition of the country among Chinese populations, as well as the positive framing of news events in Chinese journalism (Deng et al., 2023; Mert and Wang, 2025; Wang and Mert, 2025).

However, alternative interpretations remain for the unique pattern of collective future thinking observed in Chinese. Studies comparing personal and collective future thinking have typically utilized a within-subjects design, where participants are asked to imagine events to happen both to themselves and to their country (Deng et al., 2023; Mert and Wang, 2024; Shrikanth et al., 2018; Shrikanth and Szpunar, 2021). This setup may put participants in a mindset of comparison, which can raise social desirability concerns among Chinese given the strong influence of nationalism in China. Sustained by its deep historical, ethnic, and cultural roots, Chinese nationalism has risen in recent years, emphasizing sovereignty and territorial integrity of the country and promoting identity and unity among the citizens (Boylan et al., 2021; Roach, 2016; Wong, 2020). Accordingly, many Chinese individuals strongly share a “common fate” with their country and view criticisms of their country as blames to themselves personally (Dickson, 2016). Thus, when asked to think about their own future and the nation’s future side by side, Chinese participants may feel similarly optimistic about their country’s future as about their personal future and the positive outlook may “spill over” both ways.

In addition, the extent to which people believe they/their country would cause a future personal/national event to happen, namely, perceived control, may also play a role in influencing the valence of future thinking. Research has shown that perceiving greater control over future events leads people to feel more optimistic about the future (Klein and Helweg-Larsen, 2002; McElwee and Brittain, 2009; Topcu and Hirst, 2020). Given their strong tendency to attribute agency to collective groups (Markus and Kitayama, 2003; Menon et al., 1999), Chinese participants may believe that their country has greater control over its future than they do over their personal future when in a comparative mindset. The perceived control for future events may, in turn, contribute to optimism about the future.

In sum, studies to date on future thinking have mostly involved participants from Western countries, which have generally revealed a homogenous pattern of a positivity bias toward the personal future and a negativity bias toward the nation’s future (e.g., Deng et al., 2023; Shrikanth and Szpunar, 2021; Yamashiro et al., 2023). Findings with Chinese participants are important to suggest that this disassociation between personal and collective future thinking may not be universal (Deng et al., 2023). Yet additional work is required to address the alternative interpretations of the findings and confirm the unique pattern of future thinking in Chinese, independent of methodological differences. The research will enrich the theoretical understanding of the anticipatory processes in personal and collective future thinking. It will further provide valuable insights into factors that contribute to a positive outlook for the futures of people and society, which is especially critical at the current time of global challenges and geopolitical conflicts (Wang and Mert, 2025).

## The present research

The present research aims to extend the literature by utilizing a between-subjects design to investigate personal and collective future thinking in Chinese college students (Study 1) and community adults (Study 2). To address the alternative interpretations of prior findings, we compared the valence and perceived control between personal and national future events and also examined the relation between valence and perceived control within each condition. In addition, we examined the mental health implications of personal relative to collective future thinking in our mainland Chinese participants, given the practical importance of future thinking for psychological wellbeing and yet no relevant data for Chinese in prior research. Participants were randomly assigned to a personal or a national future condition, where they imagined specific future events that might happen to them or their country in 1 week, 1 year, and 10–15 years. They were asked to rate the emotional valence and perceived control of each event, and they then completed a wellbeing measure. Data collection of the studies took place in the summer through winter 2020, in the midst of the COVID-19 pandemic.

We expected that (H1) Chinese participants would imagine personal and national events similarly positive in emotional valence across all time points, but especially in the distant future, given the increasing positive outlook for the remote relative to the near future (Mert et al., 2023; Shrikanth et al., 2018). We further expected (H2) similar levels of perceived control between personal and collective future events among Chinese participants and, in line with the literature (Deng et al., 2023; Hallford et al., 2022; Topcu and Hirst, 2020), we expected higher perceived control for near than distant future events in both conditions. In addition, we expected that valence and perceived control ratings would be significantly correlated within each condition, such that participants who perceived greater control for future events would anticipate more positive future events (McElwee and Brittain, 2009; Topcu and Hirst, 2020). Finally, we expected (H3) the positivity of both personal and collective future thinking to be associated with psychological wellbeing. On the other hand, given that control and agentic goals are not emphasized in Chinese culture and therefore have reduced relevance to mental health (Cheng et al., 2013; Smith et al., 1995), we expected perceived control of either personal or national future events to have little association with wellbeing. The research material and data of the studies can be accessed at https://osf.io/dzyan.^1^

## Study 1: future thinking in Chinese college students

### Method

#### Participants

The study employed a 2 (Condition: personal vs. national) × 3 (Temporal distance: next week vs. next year vs. 10–15 years) mixed-model design, with condition as a between-subjects factor and temporal distance as a within-subjects factor. A power analysis with G*Power (Faul et al., 2007) for the 2×3 design with within-between interaction indicated that we needed 214 participants to achieve a power of 0.90 to detect effects with a size of *f* = 0.10 and *α* = 0.05. In anticipation of attrition, we recruited a total of 243 college students at Peking University, China, among whom 17 were excluded (16 failed the attention check questions—7 in the personal and 9 in the national future condition; 1 did not provide usable data). The final sample included 226 participants (*M*_age_ = 20.04 *SD* = 1.68; 47.8% female). Participants were each compensated with 10 Chinese Yuan (~$1.5).

#### Procedure

Participants were randomly assigned to a personal future (*n* = 119) or a national future condition (*n* = 107). They were instructed to imagine events that might happen to themselves (i.e., personal future) or their country (i.e., national future) at three different time points: next week, next year, and in 10–15 years. The time points were presented randomly to participants within each condition. Following prior research on episodic future thinking (Addis et al., 2008; D'Argembeau and Van der Linden, 2004; Wang et al., 2019), instructions for both conditions emphasized that the events should be specific, one-time events situated in a particular time and place, lasting no longer than a day, plausible given the individual’s or country’s future plans, and not having occurred previously. Participants were asked to imagine the events as if they were happening in their personal or country’s future and to describe the events in as much detail as possible.

After describing the events, participants rated on 7-point scales their emotional valence (i.e., “What is the overall emotional tone of this event that can happen to you/your country next week/next year/in 10–15 years?”; 1 *= very negative,* 7 *= very positive*) and perceived control (i.e., “How much do you think that you, someone else, and circumstances beyond anyone’s control may cause this event to happen next week/next year/in 10–15 years?” and “How much do you think that your country, another country, and circumstances beyond any country’s control may cause this event to happen next week/next year/in 10–15 years”; 1 = *not at all*, 7 = *very much*). Following Mert and Wang (2024), we computed a perceived self-control (personal future condition) or own country control (national future condition) score by subtracting perceived somebody else/another country control and circumstance control ratings from perceived self/own country control ratings, where higher scores indicated higher perceived self/own country control relative to other factors. Finally, participants completed the Future Time Perspective Scale and the Flourishing Scale and provided demographic information.

#### Measures

##### Future time perspective

Participants’ future orientation was measured as a potential covariate with the Future Time Perspective Scale (Carstensen and Lang, 1996). This 10-item scale assesses how expansive or limited individuals perceive their future to be, focusing on perceived time left in life, openness to future opportunities, and motivational orientation (e.g., “Many opportunities await me in the future”). Participants rated each item on a 7-point scale from 1 (*very untrue*) to 7 (*very true*). A future time perspective score was calculated by summing the ratings across the items, with higher scores indicating more open-ended future time perception. Cronbach’s alpha was 0.87 in the current sample.

##### Psychological wellbeing

Psychological wellbeing was assessed using the 8-item Flourishing Scale (Diener et al., 2010), which measures an individual’s overall functioning in life (e.g., “I lead a purposeful and meaningful life”). This scale was chosen because of its many strengths, including brevity, coverage of multiple facets of wellbeing (e.g., happiness, self-esteem, purpose, and social connection), and validity across diverse populations and languages (Bartholomew et al., 2024; Diener et al., 2010; Silva and Caetano, 2013; Sumi, 2014; Tang et al., 2016). Participants rated each item on a 7-point scale from 1 (*strongly disagree*) to 7 (*strongly agree*). A psychological wellbeing score was calculated by adding up the ratings across the items. Higher scores indicated better psychological wellbeing. Cronbach’s alpha was 0.90 in the current sample.

### Results

#### Preliminary analyses

Participants’ descriptions of future events were reviewed to ensure compliance with the instructions. Five events from the national future condition (including 2 personal events, 2 nonsensical responses, and 1 “I don’t know” response) were excluded. Independent samples *t*-tests showed that there was no significant difference in age (*M*_personal_ = 19.96, *SD* = 1.60; *M*_national_ = 20.13, *SD* = 1.78) or future time perspective (*M*_personal_ = 46.25, *SD* = 10.72; *M*_national_ = 46.14, *SD* = 10.07) between participants in the personal and national future conditions, (*t*s < 0.75, *p*s > 0.94). Gender distribution was also comparable across conditions (Personal: 43.7% female, 56.3% male; National: 52.3% female, 47.7% male), χ^2^ (1) = 1.36, *p* = 0.24. Age, gender, and future time perspective were therefore not considered further in analysis.

#### Comparisons between personal and collective future events

**Figure 1:**
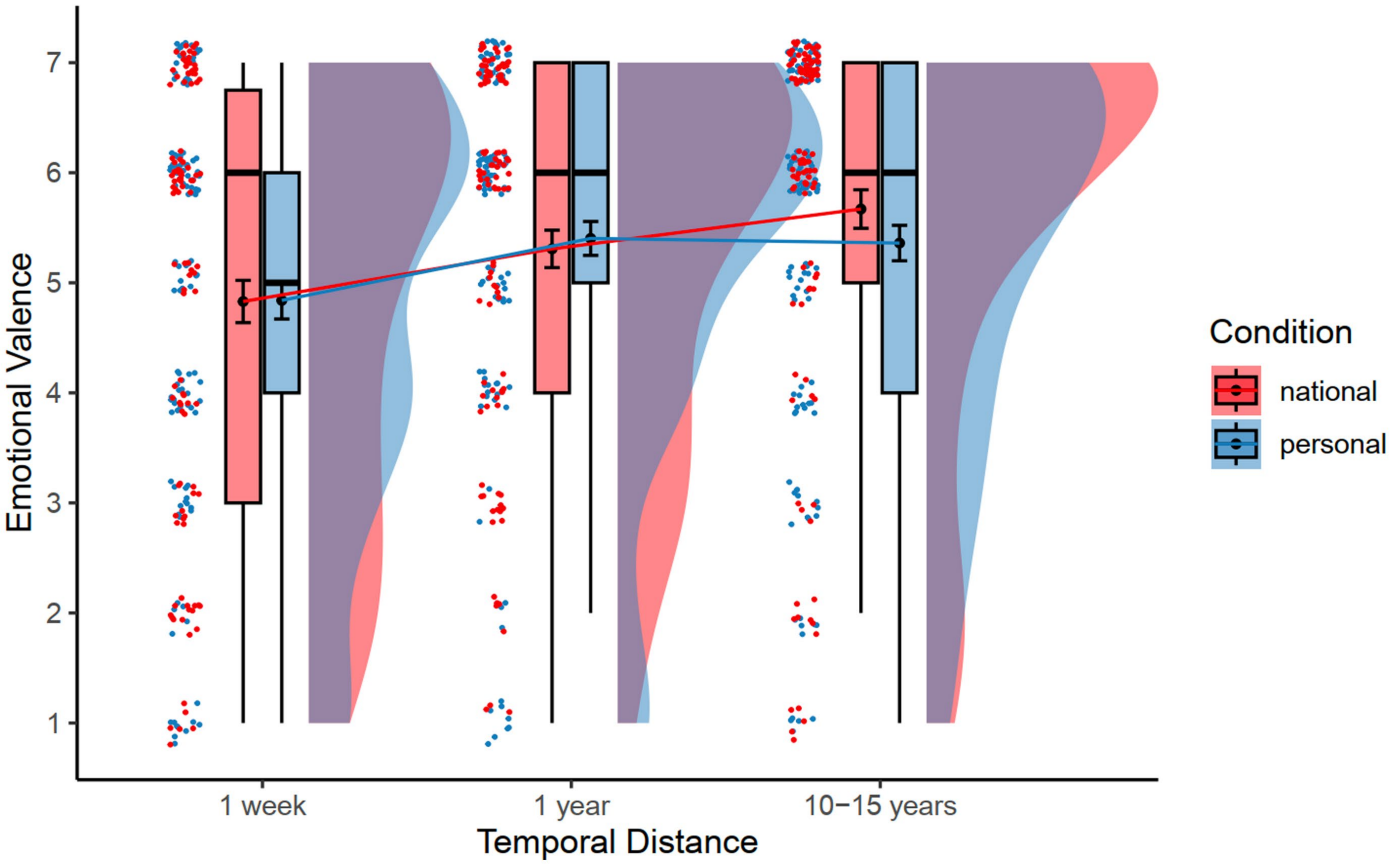
Mean emotional valence as a function of condition and temporal distance. Error bars indicate standard error of the means.

Table 1 (top panel) presents the means and standard deviations of valence and perceived control by condition and temporal distance for Study 1. Note that the mean valence scores were all positive, greater than 4 (i.e., neutral), *t*s > 4.33, *p*s < 0.001. We first conducted a 2 (Condition: personal future vs. national future) × 3 (Temporal Distance: next week vs. next year vs. 10–15 years) mixed-effects analysis on the valence ratings, with condition as a between-subjects factor, temporal distance as a within-subjects factor, and subject as a random factor. The results revealed only a main effect of temporal distance, *F*(2, 445.89) = 12.51, *p* < 0.001, η_p_^2^ = 0.05 (see Figure 1). Follow-up Tukey HSD tests (*p* < 0.05) showed that participants in both conditions imagined more positive events for the next year (*M* = 5.36, *SE* = 0.12) and for 10–15 years (*M* = 5.52, *SE* = 0.12) than for the next week (*M* = 4.83, *SE* = 0.12). There was no significant difference between events in the next year and in 10–15 years. There was no significant effect of condition or Condition × Temporal distance interaction.

**Table 1:** Means and standard deviations (*SD*) of all event variables by condition and temporal distance in Study 1 and Study 2.

	Personal future condition			National future condition		
	Week	Year	10–15 years	Week	Year	10–15 years
Study 1 (student sample)						
Valence	4.84 (1.85)	5.40 (1.68)	5.36 (1.76)	4.83 (1.97)	5.31 (1.74)	5.67 (1.80)
Perceived control	−2.74 (3.16)	−3.28 (2.87)	−3.26 (2.68)	−2.75 (3.16)	−2.27 (3.05)	−2.79 (2.72)
Study 2 (community sample)						
Valence	5.38 (1.52)	5.87 (1.45)	5.74 (1.44)	5.22 (1.78)	5.52 (1.70)	5.79 (1.46)
Perceived control	−2.97 (2.96)	−3.08 (2.91)	−3.32 (2.73)	−2.11 (3.28)	−2.66 (2.82)	−3.09 (3.12)

A similar 2 × 3 mixed-effects analysis on perceived control ratings did not reveal any significant main effect or interaction. In addition, valence and perceived control averaged across temporal distances were significantly correlated in the collective future condition, *r* = 0.31, *p* = 0.001, but not the personal future condition, *r* = 0.10, *p* = 0.29.

#### Relation to psychological wellbeing

There was no significant difference in psychological wellbeing between participants in the personal (*M* = 40.95, *SD =* 8.53) and national future conditions (*M* = 40.63, *SD =* 8.92), *t*(224) = −0.28, *p* = 0.78. Table 2 (left panel) presents zero-order correlations between psychological wellbeing and emotional valence and perceived control within each condition and temporal distance for Study 1. In the personal future condition, psychological wellbeing was positively correlated with emotional valence across all temporal distances, whereas it was not correlated with perceived control at any time point. In the national future condition, psychological wellbeing was positively correlated with emotional valence for events in the next year and in 10–15 years, as well as with perceived control of events in the next week.

**Table 2:** Correlations between wellbeing, valence, and control by condition and temporal distance for Study 1 and Study 2.

Personal future condition	Study 1	Study 2	National future condition	Study 1	Study 2
Valence					
Week	0.255^**^	0.248^**^	Week	0.179	0.008
Year	0.422^**^	0.230^*^	Year	0.320^**^	0.046
10–15 years	0.362^**^	0.250^**^	10–15 years	0.249^*^	0.253^*^
Perceived control					
Week	−0.029	−0.088	Week	0.256^**^	0.178
Year	0.086	0.070	Year	0.038	−0.030
10–15 years	0.091	−0.013	10–15 years	0.033	−0.134

*p < .05, **p < .01.

## Study 2: future thinking in Chinese community adults

### Method

#### Participants

As in Study 1, a power analysis with G*Power (Faul et al., 2007) for the 2×3 design with within-between interaction indicated that 214 participants would be needed to achieve a power of 0.90 to detect effects with a size of *f* = 0.10 and *α* = 0.05. In anticipation of attrition, we recruited 241 Chinese participants via a website similar to Amazon’s M-Turk, among whom 21 were excluded (20 failed the attention check questions—13 in the personal and 7 in the national future condition; 1 provided personal events in the national future condition). The final sample included 220 participants (*M*_age_ = 29.63, *SD* = 8.22; 63.2% female). They each received 15 Chinese Yuan (~$1.5) for their participation.

#### Procedure and measures

All procedures and measures were identical to those used in Study 1. Participants were randomly assigned to a personal future (*n* = 122) or a national future condition (*n* = 98). In the current sample, Cronbach’s alpha was 0.83 for the Future Time Perspective Scale and 0.89 for the Flourishing Scale.

### Results

#### Preliminary analyses

Based on reviews of the events descriptions, 1 event (nonsensical response) from the personal future condition and 8 events from the national future condition (6 personal events, 2 nonsensical responses) were excluded. Independent samples t-tests showed no significant difference in age (*M*_personal_ = 30.03, *SD* = 8.66; *M*_national_ = 29.13, *SD* = 7.66) or future time perspective (*M*_personal_ = 48.70, *SD* = 8.80; *M*_national_ = 49.12, *SD* = 8.08) between the two conditions (*t*s <. -81, *p*s *> 0.*42). Gender distribution was also similar across conditions (Personal: 65.6% female, 34.4% male; National: 60.2% female, 39.8% male), χ^2^(1) = 0.46, *p* = 0.50. Therefore, age, gender, and future time perspective were not considered further in analysis.

#### Comparisons between personal and collective future events

**Figure 2:**
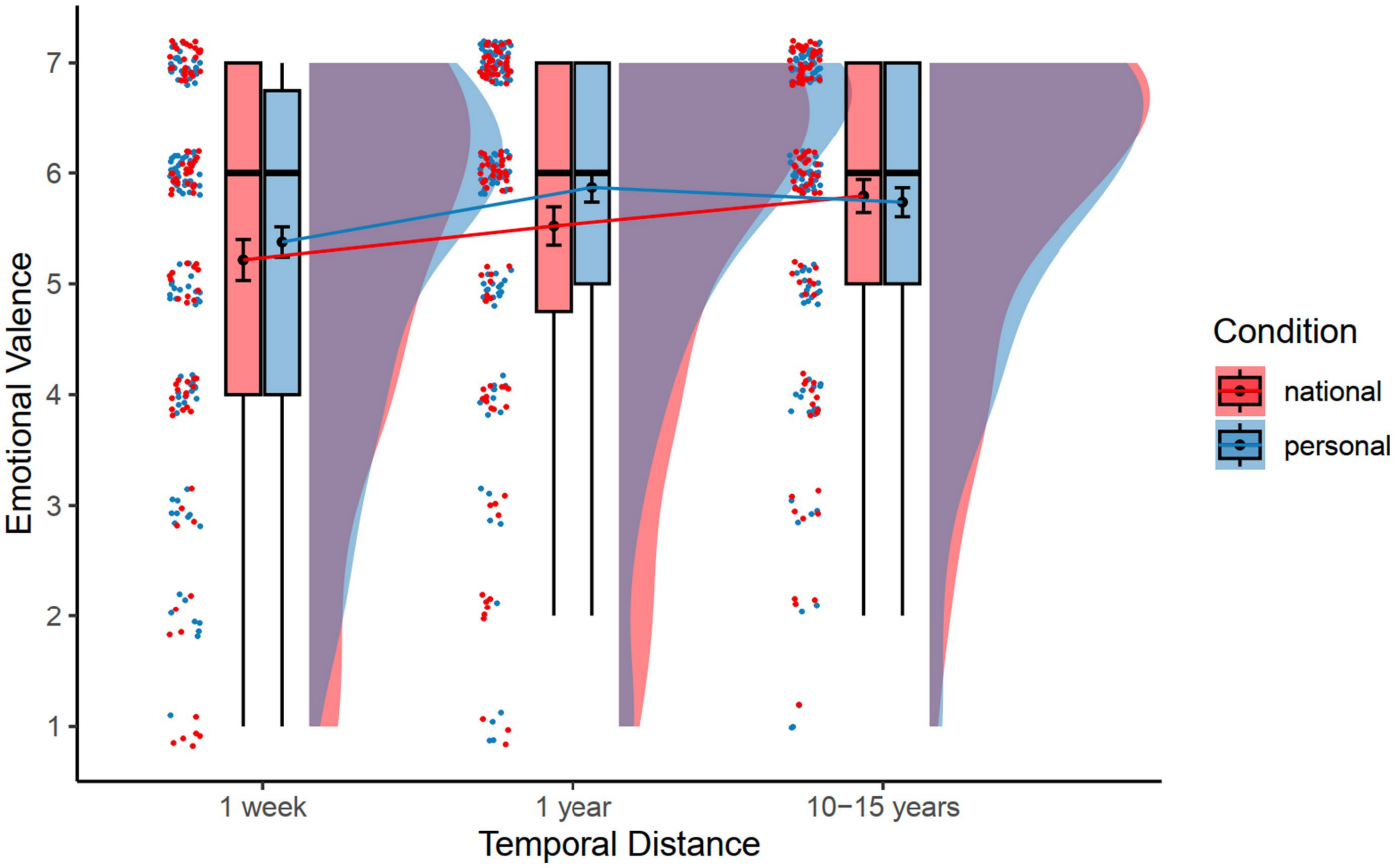
Mean emotional valence as a function of condition and temporal distance. Error bars indicate standard error of the means.

Table 1 (bottom panel) presents the means and standard deviations of emotional valence and perceived control by condition and temporal distance for Study 2. Note that the mean valence scores were all positive, greater than 4 (i.e., neutral), *t*s > 6.58, *p*s < 0.001 We conducted a 2 (Condition: personal future vs. national future) × 3 (Temporal distance: next week vs. next year vs. 10–15 years) mixed-effects analysis on the valence ratings, with condition as a between-subjects factor, temporal distance as a within-subjects factor, and subject as a random factor. The results revealed a main effect of temporal distance, *F*(2, 429.97) = 7.83, *p* < 0.001, η_p_^2^ = 0.04 (see Figure 2). Follow-up Tukey HSD tests (*p* < 0.05) showed that participants in both conditions imagined more positive events for the next year (*M* = 5.70, *SE* = 0.11) and for 10–15 years (*M* = 5.77, *SE* = 0.11) than for the next week (*M* = 5.31, *SE* = 0.11). There was no significant difference between events in the next year and in 10–15 years. There was no significant condition effect or Condition × Temporal distance interaction.

A similar mixed-effects analysis on perceived control ratings revealed a significant main effect of temporal distance, *F*(2, 430.24) = 3.54, *p* = 0.03 η_p_^2^ = 0.02. Follow-up Tukey HSD tests (*p* < 0.05) showed that participants perceived greater self/own country control for events in the next week (*M* = −2.56, *SE* = 0.20) than those in 10–15 years (*M* = −3.22, *SE* = 0.20). Events in the next year (*M* = −2.84, *SE* = 0.20) did not differ significantly in perceived control from events in either of the two other temporal distances.

In addition, valence and perceived control averaged across temporal distances were not significantly correlated in the personal, *r* = .08, *p* = .39, or collective future condition, *r* = 0.03, *p* = 0.75.

#### Relation to psychological wellbeing

There was no significant difference in psychological wellbeing between personal (*M* = 44.44, *SD =* 7.10) and national future conditions (*M* = 43.49, *SD =* 7.44), *t*(218) = −0.97, *p* = 0.33. Table 2 (right panel) presents zero-order correlations between psychological wellbeing and emotional valence and perceived control within each condition and temporal distance for Study 2. In the personal future condition, psychological wellbeing was positively correlated with emotional valence across all time points, while it was not correlated with perceived control at any time point. In the national future condition, psychological wellbeing was positively correlated with emotional valence only at the 10–15 year time point and was not significantly correlated with perceived control at any time point.

## Discussion

Understanding how people anticipate the future is of paramount importance for individual wellbeing and societal prosperity (Liu and Szpunar, 2023; Wang and Mert, 2025). Research to date on personal and collective future thinking has focused primarily on Western populations, yielding incomplete findings that may hinder and even bias the theoretical understanding of the anticipatory processes. Indeed, research with Chinese participants has suggested a unique pattern of future thinking that is different from that observed in Western populations (Deng et al., 2023; Mert et al., 2023; Yao et al., 2025). Yet only one extant study directly compared personal and collective future thinking in Chinese and the findings were subject to alternative interpretations due to the use of a within-subjects design (Deng et al., 2023). The current studies are the first to examine personal and collective future thinking in Chinese using a between-subjects design. They extend the nascent literature by comparing the valence and perceived control of personal and national future events in an experimental paradigm with random assignment and by examining the mental health implications of personal and collective future thinking in mainland Chinese participants.

Consistent with our hypotheses (H1 and H2), in both studies, participants in the personal and national future conditions imagined future events similarly positive in emotional valence across all time points, and the positivity further increased from the near future to the distant future. Furthermore, participants in both studies perceived similar levels of control by themselves over their personal future and by their country over the nation’s future. These findings suggest that the positive outlook for both personal and collective futures observed among Chinese in prior research using a within-subjects design (Deng et al., 2023) cannot be explained away by social desirability concerns or perceived group agency. When thinking about either personal or national events in the future, thus not in a mindset of implicit or explicit comparison, our Chinese student and community participants expected their country’s future to be as bright as their personal future, and the optimism further heightened for the distant future.

Many societal-cultural factors may contribute to the positive outlook of Chinese for their country’s future as much as for their personal future (Wang and Mert, 2025). Research has shown that Chinese participants exhibit a strong national identity, considering their country an important part of who they are, which is in turn associated with their anticipation of more positive and fewer negative events in the nation’s future (Deng et al., 2023; Mert et al., 2023). Furthermore, Chinese participants report general satisfaction with how things are going in their country, even during the peak of the COVID-19 pandemic^2^, which contributes to their optimism toward the nation’s future (Mert et al., 2023; Mert and Wang, 2025). In addition, news coverage in China, especially through official news outlets, tends to frame stories positively and focuses on reassuring people even in situations involving uncertainty and conflict (Chen and Koo, 2022). This may encourage positive views of the country’s current conditions and, in turn, positive expectations for its future (Mert and Wang, 2025). The present studies provide critical evidence that confirms the unique positive outlook among Chinese for the nation’s future in addition to their own. They call for further research to identify additional factors that contribute to the future-oriented optimism to inform policies and practices.

Consistent with prior findings (Deng et al., 2023; Hallford et al., 2022; Topcu and Hirst, 2020), participants in the community sample perceived higher control for near than distant future events in both personal and national future conditions. In contrast, college students perceived similar levels of control across timepoints for both personal and national futures, a finding that has also been observed in college students in the US and Turkey (Mert and Wang, 2024). Perhaps given the great personal significance that the nation’s future holds for college students’ career and life, it may seem uncertain regardless of temporal distance. Future research may investigate this possible explanation. Interestingly, valence and perceived control ratings were not significantly correlated as expected, except for collective future events in the college sample. These findings differ from prior studies based on Western samples showing that people tend to attribute greater control to themselves or their country over positive future events (McElwee and Brittain, 2009; Topcu and Hirst, 2020). They may reflect the Chinese cultural view about the natural way of life as comprising both ups and downs beyond one’s control (Markus and Kitayama, 2003; Smith et al., 1995; Wang et al., 2024). This intriguing issue begs additional research in the future.

Finally, confirming our hypothesis, across both samples of Chinese participants, the positivity of personal future thinking was associated with enhanced psychological wellbeing, consistent with previous research across different populations (Hallford and D’Argembeau, 2022; Hazan et al., 2024; MacLeod and Conway, 2007; Wang and Koh, 2015). Furthermore, the positivity of collective future thinking was also positively associated with psychological wellbeing in both samples, particularly for more distant future events, similar to findings in other populations (Hazan et al., 2024; Mert and Wang, 2024). This is consistent with the observation that when people consider—or anticipate— their country to be economically stable, environmentally safe, and socially supportive, they are happier and more satisfied with life than those who think otherwise (Pew Research Center, 2007; World Happiness Report, 2020). Yet it is important to note that the relation between future thinking and mental health is likely bidirectional (MacLeod, 2025). Interestingly, while the wellbeing of community adults was only correlated with the positivity of events in 10–15 years, the positivity of events in 10–15 years, as well as the next year, was positively associated with the wellbeing of college students. This may reflect the greater relevance of the nation’s future for college students whose personal future relies heavily on the future conditions of their country (Mert and Wang, 2024). It is therefore important in future research to consider the role of life stage in the mental health implications of personal and collective future thinking. In addition, as expected, perceived control of either personal or national future events had little association with wellbeing across both samples. This is in line with prior research showing that control and agency have limited impact on mental health in cultures where they are deemphasized (Cheng et al., 2013; Smith et al., 1995).

There are some important limitations to the current studies that should be addressed in future research. How individuals anticipate their future and the nation’s future can be influenced by the current life circumstances and societal conditions. For example, the ongoing trade war worldwide, and that between the US and China in particular, may impact individuals’ future perspectives. Our data, so as those in most studies in this area, were collected at only one specific period in time and therefore might not reflect the influence of changing circumstances. Future research may consider collecting data at multiple time points to investigate the potential shift in future thinking as a result of major events in personal life and on the national stage (Yamashiro et al., 2023). How individuals perceive change and the contributing cultural factors (e.g., analytic versus holistic thinking) may also play a role (Ji et al., 2001; Lu et al., 2025; Wang et al., 2015). Research has shown that while Chinese perceive a rising trajectory from China’s past to its future (Yao et al., 2025), Americans perceive a national decline from the US’s past to its future (Yamashiro and Roediger, 2019; Yamashiro et al., 2023). Yao et al. (2025) further observed that the more optimistic future thinking among Chinese was associated with their higher dialectical thinking when compared with Americans. More research on perceiving change and continuity in the collective cognition domain is called for. In addition, although Chinese community adults and college students showed similar patterns of future thinking in the current studies, additional cross-cohort and longitudinal studies can help to reveal how societal change and the associated psychological impacts (e.g., increased personal agency; Bain et al., 2015; Kashima et al., 2009; Kashima et al., 2011) influence individuals’ anticipation for the future.

Furthermore, the present studies did not examine specific factors that give rise to the positive outlook among Chinese for both their personal and collective futures. Although previous research has identified a host of factors that may influence the valence of future thinking (Liu and Szpunar, 2023; Wang and Mert, 2025), additional work is called for to delineate individual, cultural, and societal factors that shape how people anticipate future happenings. In addition, the between-subjects design in the current studies might have only minimized but not eliminated social-desirability pressures likely stemming from nationalism, moralized historical narratives, and self-censorship tendencies, or implicit personal–national associations due to a perceived “common fate” between self and nation. Nevertheless, the between-subjects design with random assignment avoids carryover effects between personal and collective cognitions likely to occur in within-subjects designs. Future research should consider mixed-method and interdisciplinary approaches to examine the complex anticipatory processes of personal and collective future thinking.

In conclusion, the present studies yielded original findings that Chinese participants, both college students and community adults, anticipated their country’s future to be as bright as their personal future, even when not in a comparative mindset. They also perceived similar levels of control by them over their personal future events and by their country over the national future events. The positivity of personal future events and distant national future events was further associated with psychological wellbeing. The studies demonstrate a new experimental approach to examining future thinking, and the findings inform the current understanding of anticipatory processes beyond the typical Western populations. Given the ongoing global challenges and intense geopolitical conflicts, it is critical in future research to further examine future thinking in diverse peoples and societies.

## Data Availability

The datasets presented in this study can be found in online repositories. The names of the repository/repositories and accession number(s) can be found at: https://osf.io/dzyan.

## Footnotes

1 The data on emotional valence and perceived control in collective future thinking were included in Mert et al. (2023) that addressed a different research question. All other data are original to the current article.

2
https://www.ipsos.com/sites/default/files/ct/news/documents/2020-04/what-worries-the-world-april-2020-ipsos.pdf
